# Flavonoids: Nutraceuticals for Rheumatic Diseases via Targeting of Inflammasome Activation

**DOI:** 10.3390/ijms22020488

**Published:** 2021-01-06

**Authors:** Young-Su Yi

**Affiliations:** Department of Life Sciences, Kyonggi University, Suwon 16227, Korea; ysyi@kgu.ac.kr; Tel.: +82-31-249-9644

**Keywords:** anti-inflammatory, flavonoids, inflammasomes, nutraceuticals, rheumatic diseases

## Abstract

Inflammation, an innate immune response that prevents cellular damage caused by pathogens, consists of two successive mechanisms, namely priming and triggering. While priming is an inflammation-preparation step, triggering is an inflammation-activation step, and the central feature of triggering is the activation of inflammasomes and intracellular inflammatory protein complexes. Flavonoids are natural phenolic compounds predominantly present in plants, fruits, and vegetables and are known to possess strong anti-inflammatory activities. The anti-inflammatory activity of flavonoids has long been demonstrated, with the main focus on the priming mechanisms, while increasing numbers of recent studies have redirected the research focus on the triggering step, and studies have reported that flavonoids inhibit inflammatory responses and diseases by targeting inflammasome activation. Rheumatic diseases are systemic inflammatory and autoimmune diseases that primarily affect joints and connective tissues, and they are associated with numerous deleterious effects. Here, we discuss the emerging literature on the ameliorative role of flavonoids targeting inflammasome activation in inflammatory rheumatic diseases.

## 1. Introduction

Inflammation is a biological process through which the body’s innate immune system counters the invading pathogens and senses cellular signals generated during a pathogenic invasion [1,2]. Although inflammation is a protective mechanism, chronic (repeated and prolonged) inflammation is thought to be a major causative factor for various human diseases, including inflammatory conditions, autoimmune disorders, and cancers [3,4,5,6].

An inflammatory response consists of two distinct steps, namely priming and triggering. Priming is the preparatory step, which consists of the induction of the production of inflammatory mediators, such as nitric oxide and prostaglandin E_2_, and the expression of inflammatory genes, such as tumor necrosis factor (TNF)-α, interleukin (IL)-1β, IL-6, and interferons (IFNs) [7,8,9,10]. Priming is initiated by the binding of extracellular pattern recognition receptors (PRRs), such as Toll-like receptors (TLRs), with pathogen-associated molecular patterns (PAMPs) and danger-associated molecular patterns (DAMPs) [1,2], leading to the transduction cascades of intracellular molecules in the inflammatory signaling pathways, including nuclear factor-kappa B, activator protein-1, and interferon regulatory factors [7,8,9,10]. Triggering is the stimulatory step of inflammatory responses. The cardinal feature of the triggering step is the activation of inflammasomes, intracellular multiprotein complexes consisting of intracellular PRRs, such as nucleotide-binding oligomerization domain-like receptors (NLRs), retinoic acid-inducible gene-I-like receptors (RLRs), absent in melanoma 2 (AIM2), AIM2-like receptors (ALRs), caspase-11, caspase-4, and various inflammatory molecules, such as ASC, caspase-1, and lipopolysaccharide (LPS) [11,12]. Inflammasome activation consequently induces the proteolytic activation of caspase-1, which induces the proteolytic activation of gasdermin D (GSDMD) and N-terminal fragments of GSDMD (N-GSDMD)-mediated pyroptosis via the formation of membrane pores [11,12,13,14,15]. Active caspase-1 also leads to proteolytic maturation and secretion of the pro-inflammatory cytokines IL-1β and IL-18 [11,12,13,14,15,16]. Inflammasomes are classified into two main groups, namely canonical and non-canonical. NLR family inflammasomes, including NLRP1, NLRP3, NLRC4, NLRP6, AIM2, and pyrin are canonical inflammasomes, and their regulatory roles in inflammatory responses and various human diseases have been extensively studied [17,18,19]. Non-canonical inflammasomes, mouse caspase-11 and human caspase-4/5, were recently identified and have been demonstrated to be activated in response to a unique ligand, LPS [20,21,22].

Flavonoids are a class of secondary metabolites widely found throughout the plant kingdom. Numerous flavonoids have been identified that reportedly constitute essential dietary ingredients for humans, as they increase longevity, boost immunity, and play various pharmacological roles, including anti-microbial, anti-oxidative, anti-mutagenic, cardioprotective, and anti-tumor activities [23,24]. Moreover, flavonoids have been demonstrated to possess anti-inflammatory characteristics, thereby mitigating inflammatory diseases [25,26,27]. Considerable efforts have been made to demonstrate the anti-inflammatory mechanisms and effects of flavonoids, but most of the studies have focused only on the priming step [27,28,29,30,31]. Recently, growing evidence has indicated that flavonoids also affect inflammatory diseases by targeting inflammasome activation, the main feature of the triggering step [32,33,34,35]. Rheumatic diseases are a group of disorders that primarily affect joints, connective tissues, and muscles and do not designate any specific disorder but include at least over 200 different conditions [36,37]. Many of the rheumatic diseases are considered autoimmune and inflammatory disorders, but not all rheumatic diseases are related to autoimmune and inflammatory conditions [36,37]. This is a narrative review that aims to discuss the studies investigating the pharmacological effects on rheumatic diseases of flavonoids (Table 1) targeting inflammasome activation, and also to provide new insights into the development of flavonoids as potential nutraceuticals to prevent and treat inflammatory and autoimmune diseases, including rheumatic diseases.

## 2. Inflammasome-Induced Inflammatory Responses

### 2.1. Structures and Activation of Canonical Inflammasomes

NLRP1 consists of an N-terminal PYD, followed by a nucleotide-binding and oligomerization domain (NACHT), leucine-rich repeats (LRRs), a functional-to-find domain (FIIND), and a C-terminal CARD. Bacillus anthracis toxin activates NLRP1 inflammasome by enabling interaction with NLRP1 and ASC, a bipartite adaptor, through PYD, followed by interaction with pro-caspase-1 through CARD [11,12]. Mouse NLRP1b, however, lacks an N-terminal PYF; therefore, NLRP1b directly interacts with pro-caspase-1 through CARD [11,12].

NLRP3 consists of an N-terminal PYD, followed by a NACHT, and C-terminal LRRs. The NLRP3 inflammasome is activated in response to a variety of PAMPs and DAMPs, such as ATP, cholesterol, crystals, alum, asbestos, hyaluronan, β-amyloids, bacterial pore-generating toxins, monosodium urate, silica, and pathogen-originated nucleic acid hybrids as well as biological processes, such as K^+^ efflux, Ca^2+^ influx, phagosomal rupture, oxidized mitochondrial DNA, mitochondrial damage, and reactive oxygen species (ROS). The NLRP3 inflammasome assembly is similar to that of NLRP1; the interaction of NLRP3 and pro-caspase-1 is realized through ASC [11,12].

NLRC4 consists of an N-terminal CARD, followed by a NACHT, and C-terminal LRRs. Bacterial flagellin and needle subunits activate the NLRC4 inflammasome. Interestingly, the assembly of NLRC4 is different from that of NLRP1 and NLRP3, as it does not require an adaptor ASC and it is assembled via direct interaction with pro-caspase-1 through CARD [11,12].

AIM2 consists of an N-terminal PYD and a C-terminal hematopoietic interferon-inducible nuclear protein 200 (HIN200) domain. The AIM2 inflammasome is activated in response to pathogen-derived intracellular double-stranded DNA. The assembly of the AIM2 inflammasome is similar to that of NLRP1 and NLRP3; the interaction between AIM2 and pro-caspase-1 is achieved through the adaptor ASC [11,12].

Despite the different types and activating ligands of canonical inflammasomes, they share a downstream activation mechanism. Upon the activation of inflammasomes by their ligands, the inactive pro-caspase-1 is activated by proteolytic processing. The active caspase-1, in turn, facilitates (1) the proteolytic processing of GSDMD, leading to N-GSDMD-mediated pore formation and pyroptosis, an inflammatory form of cell death, and (2) the proteolytic maturation of the inactive IL-1β and IL-18, leading to the secretion of the active forms of IL-1β and IL-18 through GSDMD pores [11,12,14].

### 2.2. Structures and Activation of Caspase-11 Non-Canonical Inflammasomes

Mouse caspase-11 and its human homologs, caspase-4 and -5, are non-canonical inflammasomes, and they share a common structure consisting of an N-terminal CARD, followed by a p20 subunit, and a C-terminal p10 subunit [38]; however, the mouse and human varieties differ in size; mouse caspase-11, human caspase-4, and -5 are 373, 377, and 434 amino acids in length, respectively. Mouse caspase-11 was first discovered as a non-canonical inflammasome, and human caspase-4 and -5 were later identified as human homologs; therefore, most of the studies on non-canonical inflammasomes have focused on the mouse caspase-11.

LPS, a pathogenic cell wall component of gram-negative bacteria, was identified as a strong agonist of caspase-11. Since gram-negative bacteria exist outside the cells, LPS derived from these bacteria must be internalized into the host cells to activate caspase-11. Several studies have demonstrated that extracellular LPS enters the host cells through receptor-mediated endocytosis. LPS interacts with CD14 and MD2, and then binds to TLR4, and the LPS/MD2/CD14/TLR4 complex delivers LPS into the host cells by endocytosis [15,39]. LPS also interacts with the hepatocyte-related high-mobility group box 1 (HMGB1) and binds to the receptor for advanced glycation end-product (RAGE), and the LPS/HMGB1/RAGE complex also delivers LPS into the host cells via endocytosis [15,40]. Interestingly, gram-negative bacteria generate outer membrane vesicles (OMVs) containing LPS, which can also be delivered into the host cells by endocytosis [15,41].

Caspase-11 non-canonical inflammasomes are activated in response to intracellular LPS by the direct interaction between CARD of caspase-11 and the lipid A moiety of LPS. Upon activation, LPS-caspase-11 complexes are oligomerized, and caspase-11 inflammasomes induce the proteolytic processing of GSDMD, leading to N-GSDMD-mediated membrane pore formation and pyroptosis [11,12,14,22]. Interestingly, recent studies have demonstrated the functional crosstalk between the caspase-11 non-canonical and NLRP3 inflammasomes during inflammatory responses. Caspase-11 activates the NLRP3 inflammasome by inducing K^+^ efflux [12,15,42,43], and this causes proteolytic activation of caspase-1, leading to caspase-1-mediated maturation and secretion of IL-1β and IL-18, and N-GSDMD-mediated pyroptosis [12,15,22,44,45], which implies that canonical and non-canonical inflammasomes cooperate to induce inflammatory responses and that there may be functional crosstalk between non-canonical caspase-11 and other types of canonical inflammasomes during these responses.

## 3. Regulatory Roles of Flavonoids in Inflammasome-Mediated Rheumatic Diseases

### 3.1. Gouty Arthritis

Gouty arthritis (GA) is a common and complex form of inflammatory arthritis that develops in people with hyperuricemia, defined as high blood levels of uric acid that form needle-like crystals within the joints and bursae. GA is characterized by severe and recurrent bouts of redness, tenderness, swelling, and pain in the joints. Recent studies have reported that the prevalence and incidence of GA vary widely depending on the countries and population studied, but the general ranges are from <1% to 6.8% and 0.58 to 2.89 per 1000 person-years, respectively [46]. Numerous studies have reported that flavonoids exert anti-inflammatory and ameliorative effects on GA by inhibiting inflammasome activation [47,48,49,50,51,52,53,54,55].

Icariin is a bioactive flavonol isolated from horny goat weed. It has been used as a Chinese herbal medicine, and diverse pharmacological characteristics of icariin have been reported, such as anti-inflammatory, anti-cardiovascular, and anti-cancer properties [56,57,58]. The anti-inflammatory activity occurs through the inhibition of inflammasome activation [59,60], and Cao investigated the ameliorative effect of icariin on GA in monosodium urate (MSU)-induced GA rats. Icariin exerted an anti-inflammatory effect and alleviated GA by suppressing ankle swelling rates, inflammatory cell infiltration, and pro-inflammatory cytokine levels in the synovial tissues of the disease rats [47]. Icariin also reduced the expression of NLRP3 inflammasome and nuclear factor-kappa B (NF-κB) pathway-related proteins in the disease rats, which relieved GA, suggesting that icariin behaved as an anti-inflammatory agent by suppressing NLRP3 and NF-κB pathways.

Procyanidins are natural polymeric flavonoids formed from catechin and epicatechin molecules, which are found throughout the plant kingdom, particularly in fruits, vegetables, grains, and nuts [61,62]. Procyanidin B2 is a phenolic compound mainly found in grapes, apples, and cocoa that has been reported to possess anti-inflammatory activity, and can exert this effect by inhibiting inflammasome activation [48,63,64]. Qiao et al. investigated the suppressive effect of procyanidin B2 on GA in MSU-induced GA mice [48]. Procyanidin B2 alleviated inflammatory responses and GA symptoms by suppressing the infiltration of inflammatory immune cells, such as macrophages and neutrophils in the air pouch and paws of the MSU-induced GA mice [48]. Moreover, procyanidin B2 decreased the expression of NLRP3 and IL-1β in the air pouch and paws of the GA mice and suppressed IL-1β release from the MSU plus LPS-stimulated mouse peritoneal macrophages (MPMs) [48]. These results suggest that procyanidin B2 alleviates GA by inhibiting NLRP3 inflammasome activation and the consequent IL-1β secretion from macrophages.

*Trans*-Chalcone is a flavonoid precursor. Staurengo-Ferrari et al. evaluated the protective effect of *trans*-Chalcone on GA in mice. *Trans*-Chalcone suppressed the MSU-induced GA symptoms, inflammatory cell recruitment, oxidative stress, and serum levels of inflammatory cytokines, such as TNF-α, IL-1β, and IL-6 in GA mice [49]. Interestingly, *trans*-Chalcone inhibited the NF-κB activation and the NLRP3 inflammasome activation by reducing the expression of NLRP3, ASC, pro-caspase-1, and pro-IL-1β, as well as IL-1β secretion in LPS-stimulated macrophages [49], suggesting that *trans*-Chalcone also inhibited NLRP3 inflammasome activation.

Hesperidin methylchalcone, a methylated hesperidin, is found in vegetables and fruits, particularly citrus fruits. Ruiz-Miyazawa et al. evaluated the therapeutic potential of hesperidin methylchalcone in the MSU-induced GA mouse model. Hesperidin methylchalcone decreased GA symptoms, leukocyte infiltration, oxidative stress, and inflammatory cytokine production in MSU-induced GA mice [50]. Hesperidin methylchalcone also decreased the expression of NLRP3, ASC, pro-caspase-1, and pro-IL-1β, inhibited the NF-kB activation, and induced the mRNA expression of the nuclear factor erythroid 2–related factor 2 (Nrf2)/heme oxygenase-1 (HO-1) in the MSU-induced GA mice [50] through NLRP3 inflammasome activation, thereby demonstrating that it could also be a potential agent for GA treatment.

Studies have demonstrated that procyanidins decrease the incidence of inflammatory diseases [65] and also inhibit NLRP3 inflammasome activation in microglia [66]. Liu et al. evaluated the effect of procyanidins on gout pain in an MSU-induced GA mouse model and macrophages. Procyanidins attenuated gout pain and mitigated gout symptoms in MSU-induced GA mice [51]. Procyanidins also inhibited the activation of NLRP3 inflammasome and caspase-1 and the production of IL-1β and ROS levels in MSU-stimulated RAW264.7 cells [51]. These results suggest that procyanidins alleviate gout pain by suppressing NLRP3 inflammasome-activated inflammatory responses in macrophages.

Morin, a dietary bioflavonoid, is present predominantly in fruits, vegetables, and grains [67] and has been reported to possess anti-inflammatory, anti-oxidative, anti-cancer, and cardioprotective properties [68,69]. Dhanasekar et al. investigated the anti-inflammatory effect of morin in MSU-induced GA rats. Morin reduced inflammation, oxidative stress, serum levels of pro-inflammatory molecules, such as TNF-α, IL-1β, IL-6, monocyte chemoattractant protein (MCP)-1, vascular endothelial growth factor (VEGF), prostaglandin E_2_ (PGE_2_), and articular elastase, and GA symptoms in MSU-induced GA rats [52], which supported findings of a previous study demonstrating the anti-inflammatory effect of morin in MSU-stimulated macrophages (RAW264.7 cells) [70]. Morin also prevented NLRP3 inflammasome activation by decreasing the expression of NLRP3 and caspase-1 and by reducing the serum level of IL-1β in MSU-induced GA rats [52]. Moreover, Morin inhibited the NF-κB activation and reduced the expression of pro-inflammatory cytokines, inflammatory enzymes, such as inducible nitric oxide synthase (iNOS), and cyclooxygenase-2 (COX-2) in GA rats. These results indicate that morin played a suppressive role in GA by inhibiting the activation of NLRP3 inflammasome and NF-κB pathways.

Epigallocatechin-3-gallate (EGCG) is polyphenol catechin abundantly present in green tea and has been demonstrated to exhibit strong anti-oxidative and radical scavenging activities [71,72]. EGCG has also been reported to possess therapeutic potential against various human diseases, including inflammatory diseases [32,73]. Jhang et al. investigated the effect of EGCG on inflammation and GA in MSU-induced GA mice and macrophages. The results demonstrated that EGCG alleviated GA symptoms by decreasing neutrophil infiltration and IL-1β secretion in the MSU-induced GA mice [53]. EGCG also prevented MSU-induced inflammation, NLRP3 inflammasome activation by reducing the expression of NLRP3 and IL-1β, and the secretion of pro-inflammatory mediators, such as IL-1β, IL-6, MCP-1, and amyloid A in the MSU-induced GA mice and THP-1 cells [53], suggesting that EGCG ameliorates GA symptoms by suppressing NLRP3 inflammasome activation and inflammatory mediators in macrophages.

Ferulic acid is a dietary polyphenol found in various plants and has been reported to demonstrate anti-inflammatory and anti-oxidative activities [74,75,76]. Doss et al. investigated the anti-inflammatory effect of ferulic acid on GA in MSU-induced GA rats and demonstrated that ferulic acid suppressed the inflammatory symptoms of GA, including paw edema, oxidative stress, and increased levels of inflammatory mediators and pro-inflammatory cytokines (TNF-α and IL-1β) in MSU-induced GA rats [54]. Moreover, ferulic acid showed anti-inflammatory activity by decreasing the expression of NLRP3 inflammasome, caspase-1, pro-inflammatory cytokines, and NF-κB in the MSU-induced GA rats [54], thereby implying that ferulic acid also alleviated GA by inhibiting the activation of NLRP3 inflammasome and NF-κB pathways.

Catechin is a naturally occurring phenolic compound abundant in tea and berries and has multiple health-promoting and disease-preventing properties due to its anti-inflammatory, anti-oxidative, and immunoregulatory effects [77]. Jhang et al. investigated the protective effect of catechin on GA in MSU-induced GA mice and macrophages. The results showed that catechin suppressed inflammation by reducing IL-1β secretion in MSU-induced GA mice [55]. Catechin also inhibited NLRP3 inflammasome activation and IL-1β secretion in MSU-stimulated THP-1 cells. Additionally, catechin reduced the mitochondrial ROS production and mitochondrial transmembrane potential impairment in THP-1 cells [55]. These results indicate that catechin was also a potential therapeutic agent for GA by suppressing NLRP3 inflammasome activation and mitochondrial damage.

**Figure 1:**
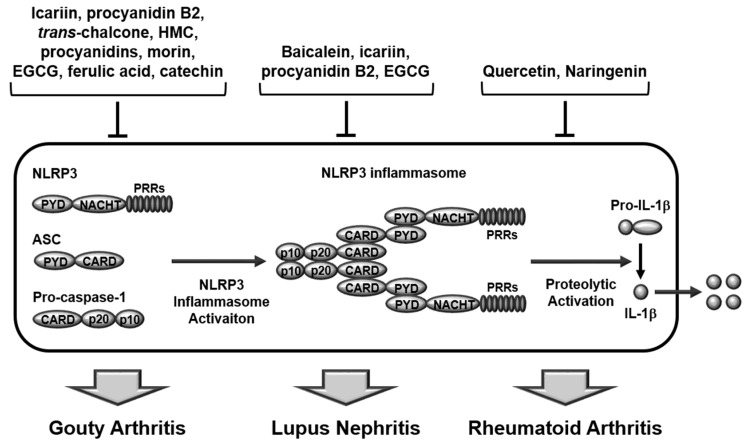
Ameliorative role of flavonoids in rheumatic diseases by inhibiting inflammasome activation. Icariin, procyanidin B2, *trans*-Chalcone, HMC, procyanidins, morin, EGCG, ferulic acid, and catechin play an anti-inflammatory role and alleviate GA in GA animal models and macrophages. Baicalein, icariin, procyanidin B2, and EGCG play an anti-inflammatory role and alleviate LN in lupus animal models and MDSCs. Quercetin and naringenin play anti-inflammatory roles and alleviate RA in RA animal and cell models. HMC, hesperidin methylchalcone; EGCG, epigallocatechin-3-gallate; GA, gouty arthritis; LN, lupus nephritis; RA, rheumatoid arthritis.

Although the above-mentioned studies successfully demonstrated a protective effect of various natural flavonoids on GA by suppressing inflammatory responses through the inhibition of NLRP3 inflammasome activation, all studies focused only on the NLRP3 inflammasome and demonstrated the protective effect of flavonoids on GA using only MSU-induced cell and animal models. Therefore, further studies are warranted to identify and validate other types of inflammasomes inhibited by flavonoids in GA pathogenesis, and to determine the protective effect of flavonoids in GA patients. The protective roles of flavonoids in GA by targeting NLRP3 inflammasome activation during inflammatory responses are depicted in Figure 1.

### 3.2. Systemic Lupus Erythematosus

Systemic lupus erythematosus (SLE), also known simply as lupus, is an inflammatory autoimmune disease in which the body’s immune system attacks the self-antigens in several locations of the host body. SLE is characterized by a red butterfly-shaped rash on the face, painful and swollen joints, hair loss, mouth ulcers, inflamed lymph nodes, tiredness, fever, and chest pain. Interestingly, most SLE patients are female (10:1 ratio), which might indicate there is a correlation between SLE and the female sex hormones [78]. Prevalence and incidence of SLE vary according to the country and ethnicity, but the overall prevalence and incidence rates range from 6.5 to 178 and from 0.3 to 23.7 per 100,000 person-years, respectively [79]. Similar to GA discussed earlier, several studies have reported that the pharmacological effects of flavonoids on SLE and SLE-induced diseases occur through the suppression of inflammatory responses by the inhibition of inflammasome activation.

Baicalein is a biophenol flavonoid present in the roots of *Scutellaria baicalensis*, and it has long been used as a Chinese traditional herbal medicine, mainly because of its anti-inflammatory, anti-oxidative, neuroprotective, and anti-cancer effects [80,81,82]. Li et al. evaluated the anti-inflammatory and anti-oxidative effects of baicalein and investigated its effect on lupus nephritis (LN) in the lupus animal model, pristine-induced lupus mice, and myeloid-derived suppressor cells (MDSCs). Baicalein suppressed inflammation and oxidative stress and reduced disease activity by attenuating proteinuria, renal function impairment, and renal histopathologies, such as cell proliferation, cellular crescents, and podocyte injury in pristine-induced lupus mice [83]. Baicalein also inhibited NLRP3 inflammasome activation by decreasing the expression of NLRP3, caspase-1, and IL-1β, the activation of NF-κB by suppressing NF-κB phosphorylation, and the levels of ROS in pristine-induced lupus mice and LPS-stimulated MDSCs [83]. Moreover, baicalein enhanced Nrf2 activation in pristine-induced lupus mice and LPS-stimulated MDSCs [83]. These results suggest that baicalein is both an anti-inflammatory and anti-oxidative flavonoid, which could aid in the treatment of LN by both inhibiting the activation of NLRP3 inflammasome and NF-κB pathways as well as inducing Nrf2/HO-1 pathway in MDSCs.

Icariin was reported to exhibit a therapeutic effect on LN by suppressing inflammasome activation in SLE. Su et al. investigated this effect in LN in MRL/lpr lupus mice. Icariin attenuated the renal disease by reducing serum levels of anti-ds DNA antibody, immune complex deposition, and macrophage infiltration in the MRL/lpr lupus mice [84]. Icariin also inhibited NLRP3 inflammasome activation by decreasing the expression of NLRP3, caspase-1, and IL-1β in the kidney and serum IL-1β levels in MRL/lpr lupus mice [84]. Moreover, icariin prevented NF-κB activation and TNF-α production in MRL/lpr lupus mice [84], indicating that icariin relieved LN by inhibiting the activation of NLRP3 inflammasome and NF-κB pathways.

Procyanidin B2 was also demonstrated to have an ameliorative effect on LN by targeting inflammasome activation in SLE. He et al. investigated this effect in LN in MRL/lpr lupus mice. The results showed that procyanidin B2 prevented renal damage and functional impairment by reducing serum levels of anti-ds DNA antibody and renal immune complex deposition in the MRL/lpr lupus mice [85]. Moreover, procyanidin B2 inhibited NLRP3 inflammasome activation by decreasing the expression of NLRP3, ASC, and caspase-1, and also reduced the renal and serum levels of IL-1β and IL-18 in the MRL/lpr lupus mice [85], suggesting that procyanidin B2 played anti-inflammatory and LN-protective roles in SLE by suppressing NLRP3 inflammasome activation and the production of IL-1β and IL-18.

EGCG was reported to demonstrate a protective effect on LN by inhibiting inflammasome activation in SLE. Tsai et al. investigated the anti-inflammatory and pharmacological effects of EGCG on LN in New Zealand black/white (NZB/W) F1 lupus-prone mice and found that EGCG prevented proteinuria, severe renal damage, and functional impairment of the kidney by inhibiting renal inflammation and oxidative stress in NZB/W F1 lupus-prone mice [86]. EGCG also inhibited NLRP3 inflammasome activation by reducing NLRP3 expression and production of active caspase-1, IL-1β, and IL-18 in NZB/W F1 lupus-prone mice [86]. Moreover, EGCG reduced NF-κB activation and increased renal Nrf2 activity in NZB/W F1 lupus-prone mice [86]. The results clearly demonstrate that EGCG had a prophylactic effect on LN associated with the suppression of NLRP3 inflammasome and NF-κB as well as the enhancement of the Nrf2 anti-oxidant signaling pathway.

All studies discussed above clearly show that numerous flavonoids exhibit anti-inflammatory, anti-oxidative, and protective effects on LN during SLE pathogenesis by targeting the NLRP3 inflammasome and NF-κB activation pathways and by inducing the Nrf2 signaling pathway. However, like GA, all studies focused only on the NLRP3 inflammasome, using only cell and animal models, which warrants further studies focusing on the evaluation of the therapeutic effect of flavonoids in human SLE patients, as well as investigation of other types of inflammasomes. Taken together, these studies clearly suggest that various flavonoids play protective and pharmacological roles in LN via deactivation of the NLRP3 inflammasome during SLE pathogenesis as depicted in Figure 1.

### 3.3. Rheumatoid Arthritis

Rheumatoid arthritis (RA) is an inflammatory autoimmune disease that primarily affects the joints, predominantly wrists, hands, and knees, and is characterized by hot, swollen, stiff, and painful joints. RA is a systemic disease that also affects other parts of the body, such as the eye, skin, muscle, and lungs. Similar to GA and SLE, the prevalence and incidence rates of RA substantially vary depending on the country and ethnicity. However, numerous studies have reported that the worldwide prevalence of RA ranges from 0.24% to 1%, and the annual incidence of RA in the United States and European countries is approximately 40 per 100,000 person-years [87]. RA is an inflammatory and autoimmune disease with high prevalence and incidence rates among rheumatic diseases, and numerous flavonoids have been demonstrated to alleviate inflammation in RA [32,88,89,90]. However, only a few studies have reported the ameliorative effect of flavonoids on RA by targeting inflammasome activation.

Quercetin is a polyphenolic flavonoid-rich in citrus fruits, vegetables, tea, berries, and red wine, and a large number of studies have demonstrated its various pharmacological attributes, such as anti-inflammatory, anti-oxidative, neuroprotective, hepatoprotective, anti-diabetic, and anti-cancer properties [32,91,92,93,94,95]. Studies have also demonstrated that the anti-inflammatory effect of quercetin can be mediated by the attenuation of inflammasome activation [96,97,98], and Yang et al. investigated this effect, and also its protective effect on RA, in collagen-induced arthritis (CIA) rats and synoviocytes. Quercetin ameliorated CIA and mitigated arthritic manifestations, such as arthritic scores, paw swelling, and increase of anti-inflammatory cytokines in CIA rats [99]. Quercetin also inhibited NLRP3 inflammasome activation by diminishing the production of NLRP3, caspase-1, and IL-1β in the synoviocytes of CIA rats [99]. Additionally, quercetin reduced the production of inflammatory mediators, such as TNF-α, IL-1β, IL-6, PGE_2_, COX-2, and iNOS enhanced by the activation of the NF-κB signaling pathway in the synoviocytes of CIA rats [99]. Interestingly, the quercetin-mediated anti-inflammatory effect was abolished by inhibition of HO-1 in the synoviocytes of CIA rats [99], indicating that Nrf2/HO-1 signaling pathways are critical for the quercetin-mediated anti-inflammatory effect in RA. These results clearly indicate that quercetin had a protective effect on RA by suppressing NLRP3 inflammasome and NF-κB activation pathways and also activating Nrf2/HO-1 signaling pathways.

Naringenin is a citrus flavonoid, predominantly distributed in various citrus fruits, other fruits, and herbs, that has also been reported to possess therapeutic potential, including anti-inflammatory, anti-oxidative, anti-bacterial, anti-viral, cardioprotective, neuroprotective, and anticancer activities [100,101,102]. A study recently reported the anti-inflammatory effect of naringenin in human diseases by targeting inflammasome activation [102]. Bussmann et al. also demonstrated its ability to suppress inflammasome activation in zymosan-induced arthritic mice. Naringenin prevented arthritic manifestations, such as articular pain, edema, inflammatory cell infiltration, tissue damage, and pro-inflammatory cytokine production in the disease mice [103]. Naringenin inhibited NLRP3 inflammasome activation by downregulating the expression of NLRP3 inflammasome components, such as NLRP3, ASC, caspase-1, and pro-IL-1β in the disease mice [103]. Naringenin also suppressed NF-κB activation and the expression of NF-κB-dependent pro-inflammatory cytokines, such as TNF-α, IL-1β, and IL-33 in the disease mice [103]. Moreover, naringenin prevented oxidative stress by inducing the expression of Nrf2 HO-1 in the disease mice [103]. These results strongly suggest that naringenin reduced inflammation and arthritic conditions by targeting the activation of NLRP3 inflammasome and NF-κB pathways as well as enhancing anti-oxidative Nrf2/HO-1 signaling pathways.

Despite a small number of studies, these two studies demonstrated the anti-inflammatory and anti-arthritic effects of naringenin by targeting inflammasome activation in different animal and cell models. However, these studies also focused only on the NLRP3 inflammasome, and therefore it is necessary to expand the research on the flavonoid-mediated anti-arthritic effects by targeting other types of inflammasomes and extend the research to clinical studies of flavonoids in RA patients. In summary, flavonoids, quercetin and naringenin play anti-inflammatory and anti-arthritic roles by preventing inflammasome activation during RA pathogenesis, as described in Figure 1.

## 4. Conclusions and Perspectives

Although inflammation is an immune response against pathogen infection and cellular stresses, chronic inflammation is regarded as a deleterious factor in a variety of human diseases. Therefore, considerable studies have elucidated molecular mechanisms of inflammatory responses with an aim to develop safe and efficacious anti-inflammatory therapeutics. As a result of these efforts, numerous anti-inflammatory drugs have been successfully developed; however, toxicity and safety issues have arisen as major critical limitations of these drugs, causing researchers to focus on the development of effective, but safer agents, such as nutraceuticals, in combination with complementary and alternative medicines.

Flavonoids are dietary bioactive nutrients found in plants, fruits, and vegetables [24], and multiple studies have demonstrated that flavonoids are safe and effective nutraceuticals that reduce inflammation and thereby act as therapeutic agents in inflammatory diseases [25,26,27]. However, most of these studies have focused on priming, a preparatory step of inflammatory responses, rather than laying focus on triggering, an activatory step of inflammatory responses. Recent studies have directed attention to the triggering step, whereby prevention of inflammasome activation evokes the flavonoid-mediated anti-inflammatory effect.

This review discusses the studies investigating the ameliorative effect of flavonoids on inflammatory rheumatic diseases, specifically the suppression of inflammasome-activated inflammatory responses. The results suggest that various flavonoids effectively assuage the deleterious effects of rheumatic diseases, such as GA, SLE, and RA by inhibiting inflammasome activation and subsequent responses, such as activation of caspase-1 and IL-1β, as summarized in Table 2.

Despite these successful studies, only a limited number of studies have evaluated the inflammasome-targeted pharmacological effect of flavonoids on human diseases. Therefore, further studies are warranted to investigate (1) the ameliorative effects of flavonoids on inflammatory rheumatic diseases through the targeting of other types of inflammasomes, particularly the caspase-11 non-canonical inflammasome for infectious diseases, (2) the targeted effects of flavonoids on inflammasome activation in non-rheumatic diseases, such as cancers and metabolic, cardiovascular, neurodegenerative, and infectious diseases, and (3) translational and clinical studies of flavonoids as nutraceuticals in human patients. In conclusion, flavonoids are effective and safe natural dietary compounds that mitigate inflammatory rheumatic diseases by inhibiting inflammasome-activated inflammatory responses and are promising nutraceuticals that possess the potential to prevent and treat rheumatic and other inflammatory diseases.

## Figures and Tables

**Table 1:** The names, groups, molecular weight (MW), and chemical formula of the flavonoids discussed in this study are listed.

Name	Group	MW	Chemical Formula
Icariin	Flavonol	676.67	C_33_H_40_O_15_
Procyanidin B2	Proanthocyanidin	578.52	C_30_H_26_O_12_
*Trans*-Chalcone	Chalcone	208.26	C_15_H_12_O
HMC	Chalcone	624.59	C_29_H_36_O_15_
Morin	Flavonol	302.24	C_15_H_10_O_7_
EGCG	Flavanol	458.37	C_22_H_18_O_11_
Ferulic acid	Polyphenol	194.18	C_10_H_10_O_4_
Catechin	Flavanol	290.27	C_15_H_14_O_6_
Baicalein	Falvone	270.24	C_15_H_10_O_5_

**Table 2:** Summary of studies discussed in this review.

Diseases	Flavonoids	Target Inflammasomes	Study Results	Experiment Models	Ref
GA	Icariin	NLRP3	Icariin alleviated GA by suppressing ankle swelling rates, inflammatory cell infiltration, and pro-inflammatory cytokine levels in synovial tissues of MSU-induced GA rats Icariin reduced the expression of NLRP3 inflammasome in synovial tissues of MSU-induced GA rats	MSU-induced GA rats	[47]
	Procyanidin B2	NLRP3	Procyanidin B2 alleviated inflammatory responses and GA symptoms by suppressing infiltration of inflammatory immune cells, in MSU-induced GA mice Procyanidin B2 decreased expression of NLRP3 and IL-1β in MSU-induced GA mice Procyanidin B2 suppressed IL-1β release from MSU plus LPS-stimulated MPMs	MSU-induced GA mice MPMs	[48]
	*Trans*-Chalcone	NLRP3	*Trans*-Chalcone reduced GA symptoms, inflammatory cell recruitment, oxidative stress, and serum levels of inflammatory cytokines in MSU-induced GA mice *Trans*-Chalcone decreased expression of NLRP3, ASC, pro-caspase-1, and pro-IL-1β and pro-IL-1β secretion in MSU-stimulated macrophages	MSU-induced GA mice BMDMs	[49]
	Hesperidin methylchalcone	NLRP3	Hesperidin Methylchalcone reduced GA symptoms, leukocyte recruitment, oxidative stress, and inflammatory cytokine in MSU-induced GA mice Hesperidin Methylchalcone decreased expression of NLRP3, ASC, pro-caspase-1, and pro-IL-1β in MSU-induced GA mice	MSU-induced GA mice Knee joint cells BMDMs	[50]
	Procyanidins	NLRP3	Procyanidins alleviated gout pain in MSU-induced GA mice Procyanidins mitigated GA symptoms in MSU-induced GA mice Procyanidins inhibited activation of NLRP3 inflammasome and caspase-1 in MSU-stimulated RAW2647 cells Procyanidins reduced production of IL-1β and ROS in MSU-stimulated RAW2647 cells	MSU-induced GA mice RAW264.7	[51]
	Morin	NLRP3	Morin attenuated inflammation and GA symptoms in MSU-induced GA rats Morin inhibited NLRP3 inflammasome activation by decreasing expression of NLRP3 and caspase-1 and serum level of IL-1β in MSU-induced GA rats	MSU-induced GA rats	[52]
	EGCG	NLRP3	EGCG decreased neutrophil infiltration and IL-1β secretion and ameliorated GA symptoms in MSU-induced GA mice EGCG inhibited NLRP3 inflammasome activation by reducing NLRP3 and IL-1β expression in MSU-induced GA mice and THP-1 cells	MSU-induced GA mice THP-1	[53]
	Ferulic acid	NLRP3	Ferulic acid suppressed inflammatory symptoms of GA in MSU-induced GA rats Ferulic acid decreased IL-1β level NLRP3 expression in MSU-induced GA rats	MSU-induced GA rats	[54]
	Catechin	NLRP3	Catechin suppressed inflammation by reducing IL-1β secretion in MSU-induced GA mice Catechin inhibited NLRP3 inflammasome activation and IL-1β secretion in MSU-stimulated THP-1 cells	MSU-induced GA mice THP-1	[55]
SLE	Baicalein	NLRP3	Baicalein suppressed inflammation and oxidative stress and ameliorated the disease activity in the pristine-induced lupus mice Baicalein inhibited NLRP3 inflammasome activation by decreasing the expression of NLRP3, caspase-1, and IL-1β in the pristine-induced lupus mice and MDSCs	Pristine-induced lupus mice DMSCs	[83]
	Icariin	NLRP3	Icariin attenuated renal disease by reducing serum levels of anti-ds DNA antibody, immune complex deposition, and macrophage infiltration in the MRL/lpr lupus mice Icariin inhibited NLRP3 inflammasome activation by decreasing the expression of NLRP3, caspase-1, and IL-1β in the kidneys, and serum IL-1β level in the MRL/lpr lupus mice	MRL/lpr lupus mice	[84]
	Procyanidin B2	NLRP3	Procyanidin B2 prevented renal damage and functional impairment by reducing serum levels of anti-ds DNA antibody and renal immune complex deposition in the MRL/lpr lupus mice Procyanidin B2 inhibited NLRP3 inflammasome activation by decreasing the expression of NLRP3, ASC, and caspase-1 in the MRL/lpr lupus mice Procyanidin B2 reduced the renal and serum levels of IL-1β and IL-18 in the MRL/lpr lupus mice	MRL/lpr lupus mice	[85]
	EGCG	NLRP3	EGCG prevented severe damage and functional impairment of the kidney by inflammation and oxidative stress in the NZB/W F1 lupus-prone mice EGCG inhibited NLRP3 inflammasome activation by reducing NLRP3 expression and production of active caspase-1, IL-1β, and IL-18 in the NZB/W F1 lupus-prone mice	NZB/W F1 lupus-prone mice	[86]
RA	Quercetin	NLRP3	Quercetin ameliorated CIA and mitigated arthritic manifestation, such as arthritic scores, paw swelling, and increment of anti-inflammatory cytokines in CIA rats Quercetin inhibited NLRP3 inflammasome activation by diminishing production of NLRP3, caspase-1, and IL-1β in synoviocytes of CIA rats	CIA rats Synoviocytes	[99]
	Naringenin	NLRP3	Naringenin alleviated inflammatory arthritis by preventing articular pain, edema, inflammatory cell infiltration, tissue damage, and pro-inflammatory cytokine production in zymosan-induced arthritic mice Naringenin downregulated mRNA expression of NLRP3 inflammasome components, such as NLRP3, ASC, caspase-1, and pro-IL-1β in zymosan-induced arthritic mice	Zymosan-induced RA mice	[103]

## Data Availability

Not applicable.

## Abbreviations

IL	Interleukin
PRR	Pattern recognition receptor
TLR	Toll-like receptor
PAMP	Pattern-associated molecular pattern
DAMP	Danger-associated molecular pattern
NLR	NOD-like receptor
RLR	Retinoic acid-inducible gene I-like receptor
AIM2	Absent in melanoma 2
ALR	AIM2-like receptor
LPS	Lipopolysaccharide
GSDMD	Gasdermin D
NACHT	Nucleotide-binding and oligomerization domain
LRR	Leucine-rich repeat
FIIND	Functional-to-find domain
OMV	Outer membrane vesicle
GA	Gouty arthritis
SLE	Systemic lupus erythematosus
LN	Lupus nephritis
RA	Rheumatoid arthritis
MSU	Monosodium urate
EGCG	Epigallocatechin-3-gallate
